# Neuroprotective effect of dimethyl fumarate in stroke: The role of nuclear factor erythroid 2-related factor 2

**Published:** 2019-07-06

**Authors:** Anahid Safari, Hamzeh Badeli-Sarkala, Mohammad Reza Namavar, Elias Kargar-Abarghouei, Neda Anssari, Sadegh Izadi, Afshin Borhani-Haghighi

**Affiliations:** 1 Stem Cells Technology Research Center, Shiraz University of Medical Sciences, Shiraz, Iran; 2 Histomorphometry and Stereology Research Center, Shiraz University of Medical Sciences, Shiraz, Iran; 3 Department of Anatomy, School of Medicine, Shiraz University of Medical Sciences, Shiraz, Iran; 4 Clinical Neurology Research Center, Shiraz University of Medical Sciences, Shiraz, Iran; 5 Department of Neurology, University of Manitoba, Winnipeg, Manitoba, Canada

**Keywords:** Stroke, Dimethyl Fumarate, Middle Cerebral Artery, Nuclear Factor Erythroid 2-Related Factor 2

## Abstract

**Background:** There is evidence that supports the neuroprotective effects of dimethyl fumarate (DMF) in stroke. Nuclear factor erythroid 2-related factor 2 (Nrf2) has both anti-oxidant and anti-inflammatory mechanisms. We investigated the neuroprotective effects of DMF via Nrf2 activation in the cortex, striatum, and diencephalon in a middle cerebral artery occlusion (MCAO) model of stroke.

**Methods:** 22 Sprague-Dawley male rats were randomized into 3 groups. In DMF-treated group (n = 8), rats received 15 mg/kg oral DMF twice daily by gavage from day 0 to 14 after a 60-minute MCAO. The vehicle group (n = 7) underwent MCAO and were given methocel/H_2_O, using the same method and schedule. In the sham group (n = 7), neck was opened, but neither middle cerebral artery (MCA) was occluded nor any drug was administered. After 14 days, the animals were sacrificed. The infarct volume were assessed by stereology method. Nrf2 expression was evaluated in the cortex, striatum, and diencephalon by immunohistochemistry method.

**Results:** Ratio of infarct to total brain volume was significantly lower in the DMF-treated group (5.76%) in comparison with the vehicle group (22.39%) (P < 0.0001). Nrf2 expression was higher in DMF-treated group in comparison with both the vehicle and sham groups in cortex, striatum, diencephalon, and total brain (P < 0.0001). In the DMF-treated group, significant negative correlation between Nrf2 expression and infarct volume was observed in cortex, striatum, diencephalon, and total brain.

**Conclusion:** DMF induces Nrf2 expression and its neuroprotective effects in different brain anatomical regions.

## Introduction

Stroke is a prevalent cause of mortality and disability around the world.^1^ Intravenous thrombolysis and mechanical thrombectomy have great impact on the treatment of acute ischemic stroke (AIS), but with narrow therapeutic windows.^2^ Hence, there is a fundamental need to search for new treatments. Several inflammatory cells and mediators are involved in the pathogenesis of ischemic stroke.^3^ Reactive oxygen species (ROS) increases after ischemic stroke, leading to extensive cell death. Furthermore, reperfusion injury due to clot lysis and elevation in tissue oxygenation leads to a second rise in ROS production.^4^

Nuclear factor erythroid 2-related factor 2 (Nrf2) is one of the most important endogenous protective factors against oxidative stress. Nrf2 is mainly located in the cytoplasm, but it relocates to the nucleus in oxidative stress and binds to specific deoxyribonucleic acid (DNA) sites to induce the transcription of cytoprotective genes. ROS produced in both arterial occlusion and reperfusion phase of injury,^5^ are strong inducers of Nrf2.^6^

Nrf2 activation might have anti-inflammatory effects via interaction with nuclear factor kappa-light-chain-enhancer of activated B cells (NF-κB).^7^ Accordingly, there is an increasing clinical interest in using Nrf2 activators.^8^^,^^9^

Fumaric acid esters including dimethyl fumarate (DMF) and monomethyl fumarate (MMF) have been used to treat psoriasis as an immune-mediated skin disease.^10^^,^^11^ Also, they have been approved for the treatment of multiple sclerosis (MS).^12^ Middle cerebral artery occlusion (MCAO) by intraluminal thread method is the most commonly-used and reliable model that is closest to human ischemic stroke due to imitation of both ischemia and reperfusion injuries.^13^ Recently, neuroprotective effects of DMF were investigated in MCAO models of stroke due to its anti-inflammatory and cytoprotective mechanisms of action.^14^^-^^16^ It has been proposed that DMF has anti-inflammatory and anti-oxidant neuroprotective mechanisms via Nrf2 activation.^16^

In the present study, we assessed Nrf2 expression, infarct volume, and their correlation after MCAO in DMF-treated, vehicle, and sham groups. We mainly focused on Nrf2 expression in different brain anatomical regions in both affected and unaffected hemispheres.

## Materials and Methods

Total of 22 Sprague-Dawley male rats were randomly assigned into three groups by block randomization. The DMF-treated group (n = 8) underwent MCAO by intraluminal thread using Koizumi’s method.^17^ Briefly, common carotid artery (CCA) was separated from its sheath, then two loose collar ligatures were performed around the proximal and distal parts of the CCA. External carotid artery (ECA) was ligated by ligature. Permanent ligation by ligature around the proximal part of the CCA was also prepared. Blood flow into CCA just before its bifurcation was stopped temporarily by a vessel clip. Then, a small incision was made between proximal ligature and the clip. A blunted silicon-coated 4-0 nylon ligature was inserted into CCA and then through the internal carotid artery (ICA) towards the middle cerebral artery (MCA). The filament was fixed by tight ligatures. To induce stroke, the nylon ligature was kept for 60 minutes. Reperfusion was performed by removing the nylon ligature, then CCA ligature was tightened.^17^ As soon as the animals recovered from anesthesia, the first dose of DMF was administered. This group were treated with oral administration of 15 mg/kg DMF twice daily by gavage from day 0 to 14 post-operation. We used drug dose of 15 mg/kg according to previous works.^18^ A 14-day duration is the feasible period of time for rat model of stroke which can mimic both acute and subacute phases of human stroke. This long study period is not frequently planned because of difficulties in caring animals and drug administration as well as post operation complications. 

The vehicle group (n = 7) underwent MCAO and were treated with 200 µl of 0.08% methocel/H_2_O by gavage, similar to the DMF-treated group schedule. In the sham group (n = 7), neck was opened, but neither MCA was occluded nor any drug was administered. After 14 days, the rats were anesthetized with halothane and transcardially perfused with normal saline followed by 4% buffered paraformaldehyde (PFA). The brain was removed and placed in the same fixative overnight and transferred to 30% sucrose (Sigma, St. Louis, MO, USA) in a phosphate-buffered saline (PBS) for 48 hours. The frozen brain was sliced, using a cryostat (SLEE, Frankfurt, Germany) and kept in cryoprotectant solution at 20 °C. Ratio of infarct to total brain volume was evaluated by stereology method according to Cavalieri’s principle,^8^ in different brain regions (cortex, striatum, and diencephalon).

***Immunohistochemistry protocol:*** Brain sections (30 µm thickness) were washed with PBS and fixed by PFA 4% for 20 minutes. Then, immunohistochemistry evaluation was performed with primary and secondary antibodies.^19^ Nuclear counterstaining was performed with 4′,6-diamidino-2′-phenylindole dihydrochloride (DAPI). Finally, the stained sections were examined under fluorescence microscope by a researcher who was blinded to the experimental condition. Sections from identical ischemic brain regions from each group (n = 6 sections from each brain) were used for cell quantification. The number of immunopositive cells in each section were counted in 5 microscopic fields (under ×200 magnification) throughout the similar regions of the brain. The mean visual field was calculated and the immunopositive cell images were merged to DAPI-stained cell images by CellProfiler software (Broad Institute, Cambridge, MA, USA), and the percentage of Nrf2 expression was reported.

Slides were washed in PBS plus 0.025% Triton X-100 (TX100) (Sigma, St. Louis, MO, United States), then blocked in 10% normal serum (Sigma, St. Louis, MO, United States) with 1% bovine serum albumin (BSA) (Sigma, St. Louis, MO, United States) in PBS for 2 hours at room temperature. Sections were then incubated with primary antibody anti-Nrf2 (1/100, ab137550, Abcam, UK) for overnight at 4 °C.

Next, sections were incubated with fluorophore-conjugated secondary antibody [Alexa Fluor 488 goat anti-rabbit IgG secondary antibody (1/250, ab150077, Abcam, UK)] for 1 hour at room temperature. 

DAPI stock solution was diluted to 300 nM in PBS and approximately 300 µl of this solution was added to the stained preparation, incubated for 1-5 minutes, and then washed. After mounting, the brain sections were coverslipped and examined under fluorescence microscope (Olympus, Tokyo, Japan) with appropriate filters.

***Cell profiling:*** Nrf2 presentation was calculated using CellProfiler 2.1.1 software. CellProfiler is a software for biological image-based data analysis, validated for a wide variety of biological applications.^20^

***Ethics:*** The study was conducted in accordance with the guidelines of the local Ethics Committee of Shiraz University of Medical Sciences, Shiraz, Iran, and standard principles for laboratory animal care.

Statistical analysis was performed with SPSS software (version 21, IBM Corporation, Armonk, NY, USA). All data were expressed as mean and confidence interval (CI). Analysis was performed using one-way analysis of variance (ANOVA) followed by Tukey’s multiple comparison test. Pearson correlation tests were used whenever appropriate. P-value < 0.05 was considered to be statistically significant.

## Results

**Figure 1:**
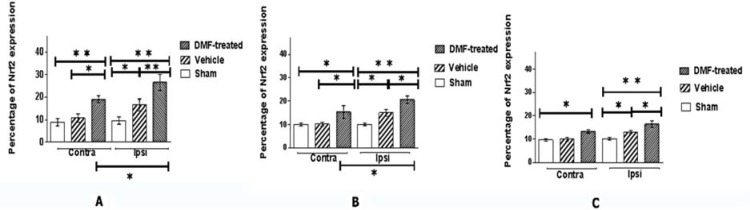
Mean percentage and confidence interval (CI) (error bars) of nuclear factor erythroid 2-related factor 2 (Nrf2) expression in the cortex (A), striatum (B), and diencephalon (C) regions in the dimethyl fumarate (DMF)-treated, vehicle, and sham groups in hemispheres ipsilateral (Ipsi) and contralateral (Contra) to middle cerebral artery occlusion (MCAO)

^*^Indicates significant differences (P < 0.05) between the DMF-treated, vehicle (A: Contra, B: Contra and Ipsi, C: Ipsi) and sham groups (B and C: Contra) as well as the vehicle and sham group (A, B, and C: Ipsi). It also indicates the significant differences between hemispheres in the DMF-treated group (A and B). ^**^Indicates significant differences (P < 0.0001) between the DMF-treated, vehicle (A: Ipsi), and sham groups (A: Contra and Ipsi, B and C: Ipsi).

Nrf2 expression mean percentage in total brain of DMF-treated, vehicle, and sham groups were 37.15%, 25.21%, and 19.49%, respectively. Figure 1 shows the mean percentage and CI of Nrf2 expression in the ipsilateral (ischemic-affected) and contralateral (unaffected) hemispheres in different brain regions of DMF-treated, vehicle, and sham groups.

Nrf2 expression in the cortex, striatum, diencephalon, and total brain was significantly different between groups (P < 0.0001 and F-values = 59.43, 31.75, 28.55, and 127.84, respectively).

Nrf2 expression was higher in DMF-treated group in comparison with the vehicle and sham groups in the cortex, striatum, diencephalon, and total brain (P < 0.0001).

Nrf2 expression was also higher in the vehicle group in comparision with the sham group in the cortex, striatum, diencephalon, and total brain (P = 0.0005, P = 0.0349, P = 0.0470, and P < 0.0001, respectively). 

Comparing the DMF-treated and vehicle groups, Nrf2 expression was not only significantly different in the affected hemisphere [the cortex (P = 0.0001), striatum (P = 0.0271), and diencephalon (P = 0.0213)], but also in the cortex (P = 0.0021) and striatum (P = 0.0265) of the unaffected hemisphere.

Significant differences were observed between the vehicle and sham groups (P = 0.0242, 0.0236, and 0.0222 for cortex, striatum, and diencephalon, respectively) as well as sham and DMF-treated groups (P < 0.0001) in the affected hemispheres. 

Nrf2 presentation was significantly increased in ipsilateral side in comparison with contralateral side in the cortex (P = 0.0092) and striatum (P = 0.0369), but not in diencephalon (P = 0.0967) of the DMF-treated group. In the vehicle group, there were no significant differences between ipsilateral and contralateral side in any of the brain regions (P > 0.0500). These data were similar in the sham group for all the mentioned regions.

**Figure 2:**
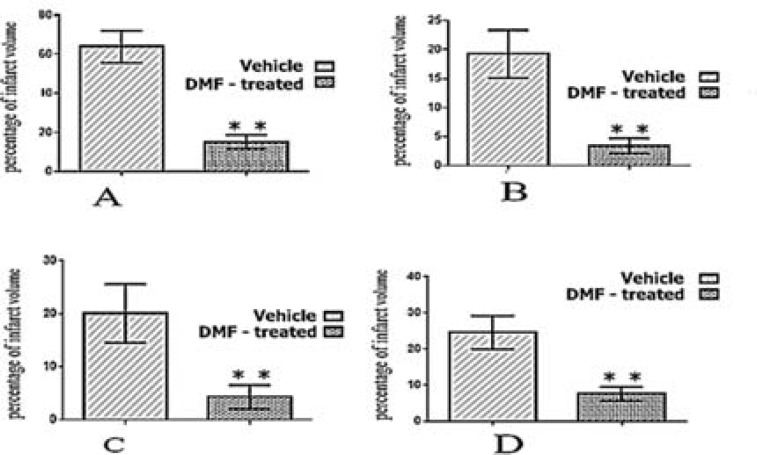
Mean percentage and confidence interval (CI) (error bars) of infarct volume in total brain (A), cortex (B), striatum (C), and diencephalon (D) regions in the dimethyl fumarate (DMF)-treated and vehicle groups

^**^P < 0.0001

As figure 2 shows, mean infarct volume in total brain and three different brain regions in the DMF-treated group was significantly smaller than vehicle group.

**Figure 3:**
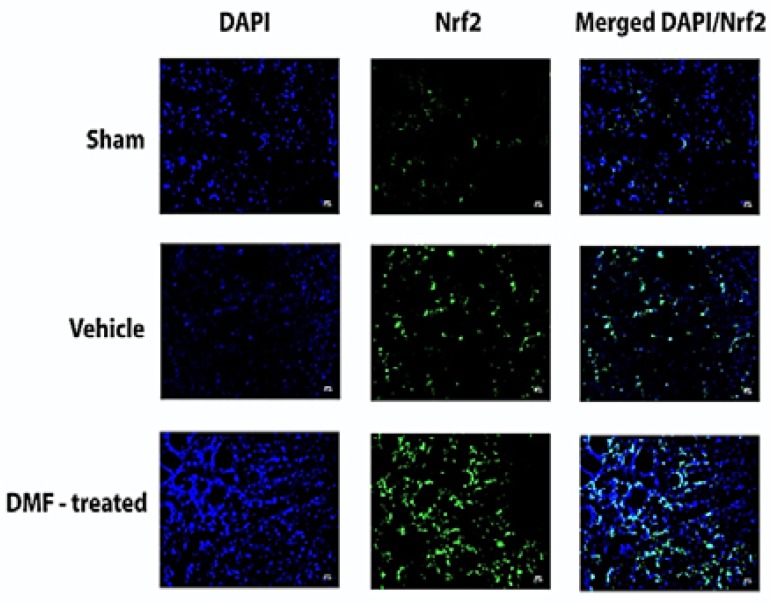
4′,6-diamidine-2′-phenylindole dihydrochloride (DAPI) for nuclear staining (blue), nuclear factor erythroid 2-related factor 2 (Nrf2) (green), and merged DAPI/Nrf2 in the cortex of sham, vehicle, and dimethyl fumarate (DMF)-treated groups; the increased number of nuclei and Nrf2 expression can be seen in the DMF-treated group in comparison with vehicle group.

Significant negative correlation was observed between the percentage of Nrf2 expression and infarct volume in the cortex (P = 0.0190, r = -0.598), striatum (P = 0.0030, r = -0.717), diencephalon (P = 0.0090, r = -0.654), and total brain (P < 0.0001, r = -0.839). Figure 3 shows DAPI and Nrf2 stained as well as DAPI/Nrf2 stained merged images in the cortex of sham, vehicle, and DMF-treated groups, indicating neuroprotective effect of DMF via Nrf2 activation.

## Discussion

Our results showed significant increase in Nrf2 presentation in the DMF-treated group in comparison with the sham and vehicle groups. Ratio of infarct volume was significantly reduced in the DMF-treated group in comparison with the vehicle group.

Significant negative correlation between the percentage of Nrf2 presentation and infarct volume was observed. 

Our results are in line with previous studies with respect to the protective effects of Nrf2 in ischemia/reperfusion brain injuries.^5^^,^^6^^,^^21^

Lin et al. showed the anti-oxidative effects of DMF in the acute phase of stroke (72 hours after MCAO) by Nrf2 and Heme oxygenase-1 (HO-1) overexpression. They also showed the anti-inflammatory effects of DMF by reducing the infiltration of inflammatory cells in the infarct area 7-14 days after inducing stroke. Abolished neuroprotective effect of fumaric esters in Nrf2^-/-^ mice was also shown in their study. They also reported that during ischemic condition, Nrf2 knockdown in endothelial cells diminished the protective effect of DMF by provoking subcellular delocalization of tight junction proteins.^14^

Clausen et al. showed that single intravenous bolus of MMF increased Nrf2 level 6 hours after MCAO. They also stated that MMF treatment did not influence infarct size, but reduced edema at both 24 and 48 hours after MCAO.^22^

Yao et al. showed that DMF and MMF decreased neurological deficits, glial activation, infarct volume, brain edema, and cell death in ischemia-induced brain injury. The neuroprotective effects of DMF and MMF were rarely seen in Nrf2^-/-^mice on the 7^th^ day post-injury.^23^

Although fumaric esters Nrf2-mediated mechanisms of action in stroke have gained much interest in recent years, only few studies focused on separate brain anatomical regions and hemispheres. In the current study, significant differences in ipsilateral side of the brain were observed separately between groups in the cortex, striatum, and diencephalon. In addition, significant differences were observed between the vehicle and DMF-treated group in unaffected hemispheres of the cortex and striatum, as well as sham and DMF-treated group in all brain regions. There was no significant difference between unaffected hemisphere of the sham and vehicle groups in any of the regions. Nrf2 presentation significantly increased in ipsilateral side in comparison with contralateral side in the cortex and striatum of the DMF-treated group in comparison with sham and vehicle groups (Figure 1).

Nrf2 can be normally expressed by different cells and tissues, which explains its minimal expression in the sham group (Figure 3: Nrf2 stained images). Stressful conditions facilitate Nrf2 translocation into the nucleus, which explains its overexpression in the vehicle group, particularly in comparison with the sham group. Significant differences between the sham and DMF-treated group in addition to vehicle and DMF-treated group (especially in stroke-affected hemispheres) verify the stronger effects of DMF in inducing Nrf2 versus the role of stroke induction per se. Stroke activated Nrf2 expression in the affected hemisphere of all regions, but DMF-inducing effect was more remarkable in both hemispheres of brain regions that were severely affected.

Nrf2 activation in the contralateral hemisphere after MCAO, which was observed in the current study, also was reported by Dang et al.^24^ They explained Nrf2 activation in the contralateral side by two hypothesis: a probable pathway of Nrf2 activation independent from ROS production and/or cross-hemispheral neural connections.^25^ Regarding ROS-independent Nrf2 regulatory mechanisms, they suggested the involvement of typical ischemic-induced cytokines and chemokines that activate microtubule-associated protein (MAP) kinase signaling pathway.

In the current study, significant differences between the sham and DMF-treated group in addition to vehicle and DMF-treated group in the contralateral hemispheres (except for diencephalon) might imply the stronger effect of DMF in inducing Nrf2 activation in comparison with MCAO per se independent from each of the hypothetical mechanisms. 

As a point of strength, we compared the neuroprotective effect of DMF via Nrf2 expression not only in the whole brain, but also in three separate regions. We also assessed both the affected and non-affected hemispheres. 

This study has some limitations: core and penumbra were not assessed separately, and Nrf2 expression in different cell types, such as astrocytes, microglia, and neurons was not evaluated. Also, cytoplasmic and nuclear localization of Nrf2 expression was not differentiated.

## Conclusion

Our results reasserted the significant role of Nrf2 in pathogenesis of subacute ischemic stroke with a focus on separate brain regions and hemispheres. Since DMF safety was described in previous studies, it is recommended to evaluate DMF in phase III human stroke researches.

## Notes:

**How to cite this article:** Safari A, Badeli-Sarkala H, Namavar MR, Kargar-Abarghouei E, Anssari N, Izadi S, et al. Neuroprotective effect of dimethyl fumarate in stroke: The role of nuclear factor erythroid 2-related factor 2. Iran J Neurol 2019; 18(3): 108-13.
